# Nonintrusive Fine-Grained Home Care Monitoring: Characterizing Quality of In-Home Postural Changes Using Bone-Based Human Sensing

**DOI:** 10.3390/s20205894

**Published:** 2020-10-18

**Authors:** Sinan Chen, Sachio Saiki, Masahide Nakamura

**Affiliations:** 1 Graduate School of System Informatics, Kobe University, 1-1 Rokkodai-cho, Nada, Kobe 657-8501, Japan; sachio@carp.kobe-u.ac.jp (S.S.); masa-n@cs.kobe-u.ac.jp (M.N.); 2 RIKEN Center for Advanced Intelligence Project, 1-4-1 Nihonbashi, Chuo-ku, Tokyo 103-0027, Japan

**Keywords:** home care monitoring, physical activity, human sensing, postural changes, pose estimation, pose conversion, body movement, positional changes

## Abstract

In contrast to the physical activities of able-bodied people at home, most people who require long-term specific care (e.g., bedridden patients and patients who have difficulty walking) usually show more low-intensity slow physical activities with postural changes. Although the existing devices can detect data such as heart rate and the number of steps, they have been increasing the physical burden relying on long-term wearing. The purpose of this paper is to realize a noninvasive fine-grained home care monitoring system that is sustainable for people requiring special care. In the proposed method, we present a novel technique that integrates inexpensive camera devices and bone-based human sensing technologies to characterize the quality of in-home postural changes. We realize a local process in feature data acquisition once per second, which extends from a computer browser to Raspberry Pi. Our key idea is to regard the changes of the bounding box output by standalone pose estimation models in the shape and distance as the quality of the pose conversion, body movement, and positional changes. Furthermore, we use multiple servers to realize distributed processing that uploads data to implement home monitoring as a web service. Based on the experimental results, we conveyed our findings and advice to the subject that include where the daily living habits and the irregularity of home care timings needed improvement.

## 1. Introduction

As the global population is aging at an accelerated rate, the number of the people requiring care increases every year. In countries with significant aging such as Japan, people are gradually shifting from facility care to home care owing to limited availability of facilities and human resources [1,2]. Continuing in-home physical activities important after leaving facility care, to extend life span [3]. In conventional approaches to evaluating physical activities, it is common to use the doubly labeled water (DLW) method [4,5], wearable sensors [6,7,8], and question-based activity assessments [9,10]. However, for people requiring special care, in-home physical activities are often limited by diseases. With the help of family caregivers, they can only maintain the most basic activities of daily living instead of physical exercise. Since invasiveness to daily living and body, individual differences, and ambiguous activity times in the existing approaches, monitoring the current detailed status of people requiring special care in real time is hard to realize.

Postural changes as a result of physical activity were introduced in [11,12]. For balance impaired and older adults, low levels of physical activity effects on physical stability. Our interest is to incorporate bone-based human sensing technologies into postural changes monitoring at home. With the advent of open-source libraries such as Tensorflow [13], various pre-trained models base on machine learning have been widely using in different fields. These models can conduct feature extraction directly at the edge of devices without network connectivity. Typical models include image classification [14,15], object detection [16,17], and pose estimation [18]. Among the various available models [19,20,21], as one of bone-based human sensing technologies, there is a standalone and trained model called PoseNet [22]. A PoseNet Model receives an image that contains a person, then returns specific information of 17 body keypoints in JavaScript Object Notation (JSON) [23]. Existing studies for human pose estimation have become a rapidly growing field [24,25].

The goal of this paper is to develop a nonintrusive fine-grained home monitoring system for people requiring specific care. To achieve the goal, We are currently investigating techniques that integrate inexpensive camera devices and standalone models of pose estimation. More specifically, we capture images of the fixed-point home space and extract the body keypoints and bounding box features with pose estimation models in real time, to accumulate specific information as time-series data. In this study, the main difference from the existing research is that using feature values of the bounding box instead of 17 body keypoints for characterizing the postural changes. We use the term “quality” of in-home postural changes in the title to represent a more detailed status, which includes not only the number of changes, but also the current pose map, such as the changes during standing up, sitting down, and walking at home. We regard the changes of the bounding box outputted by standalone pose estimation models (i.e., PoseNet Model) in the shape and distance as the quality of the pose conversion, body movement, and positional changes, which we convert to the visual charts. Moreover, we develop a web service to draw the pose maps automatically using updated 17 body keypoints once per second. The pose maps either past or present can be viewed from the browser of various devices.

The main contribution of this paper is to obviate the device intrusiveness to the daily living of users and to implement stable continuous feature data extraction in such short time intervals. Unlike the conventional approaches using high-cost dedicated device and cloud computing resources, we provide a local process that extracts the feature data from the Base64 encoding of original images [26]. The point to keep in mind is to limit it that only allows the feature data to upload through the network into the database. Accumulating small size of feature data in the storage space of the database continuously instead of original images, they improve the leakage of personal privacy data. Considering that the operability of the elderly people in installing and maintaining devices is existing, we extend the function of data acquisition from a computer browser to a “plug and play” Raspberry Pi system [27]. Users can choose a suitable device to connect to camera devices for home care monitoring based on individual requirements. We use multiple servers to realize distributed processing that uploading data to implement home monitoring as a web service. Users can use Internet browsers to access the data at any time. Besides, the proposed method also has a low-cost advantage, which can be to apply even children and adults requiring special care.

The system evaluation has performed from the accuracy of evaluating data, usage status of memory, and CPU. As the main results, the accuracy of ResNet50 is over 70% in the range of the difference value of less than ten, the usage rate of system memory and CPU respectively is around 30% and 70% in the Raspberry Pi. Based on the proposed method, we have experimented in a fixed-point home space of an elderly household for ten days. We used a PoseNet model for single-person pose estimation in a Raspberry Pi device and a camera to characterize the quality of in-home postural changes of the elderly persons requiring care. The main experimental results show that we could estimate the daily living habits of the subject and the irregularity of home care timings, and the postural changes induced by external factors such as doctor home visits. The results validate the usefulness of the proposed method. The remainder of this paper is organized as follows: In Section 2, we discuss related works on home care monitoring. We provide a detailed description of the proposed model in Section 3 and the system evaluation in Section 4. An actual experiment of evaluating in-home postural changes for home care monitoring is presented in Section 5, followed by the conclusions in Section 6.

## 2. Related Work

Implementing high-performance home care monitoring systems for the elderly has been investigated thoroughly in smart homes [28,29,30,31,32,33]. Table 1 shows a comparison of related work for home monitoring. We consider the following three challenges in implementing continuous home care monitoring: (1) Extracting informative data with a lightweight process is often hard to realize. (2) Real-time analysis using a large number of computing resources required is unrealistic. (3) The possibility of leakage of information cannot be ignored when uploading data through the network as demonstrated in [34,35,36]. In this section, we introduce some related works from recent years.

Pervasive smart home service is inseparable from lightweight processing, even during the early and late stages. Kim et al. [37] proposed a depth video-based human activity recognition (HAR) system to analyze the daily activities of elderly people in indoor environments. In [38], a method of monitoring the daily activities of elder people using commercial sensors to register recognizable activities was proposed. Although the daily activities of users can be evaluated at home from different angles, preliminary works such as algorithm design and model training still take time to be implemented in houses. We aim to identify common requirements across households for characterizing the quality of postural changes, making smart home monitoring as simple as possible. Al-Khafajiy et al. [39] presented a smart healthcare monitoring system capable of observing elderly people remotely. In [40], an elderly health care (EHC) system for covering the real health needs of the elderly was introduced. Arshad et al. [41] present a model based on daily activities that can be to monitor the mobility parameters. These smart services use diversified sensing devices that realize rich functions concerning the daily health of the elderly at home. However, we consider that there is a possibility of invasiveness to daily living and body during data acquisition based on user operation or the devices wearing.

The costs of smart homes is also a matter of concern for most users. Chiridza et al. [31] discuss the development of a low-cost smart home environment (SHE) for the elderly living independently. In [42], a design to continuously monitor the daily behaviors of the elderly in the living environment at residential homes is presented. The use of low-cost sensor devices to extract data about environments and users at home is effective. Contrary to this approach, we focus on the development of human-centric smart home services. This is advantageous in the centralized extraction and lightweight processing of key data under specific conditions. Bassoli et al. [43] proposed a new system architecture suitable for human monitoring based on Wi-Fi connectivity. In [44] has been proposed a toolkit comprising off-the-shelf, affordable sensors to monitor meaningful activities. Lee et al. [45] attempted to construct a monitoring system for elderly people living alone. Using several sensor devices to realize smart homes, involves factors such as irregular changes in the placement of each device, and the long-term maintenance of various devices. In this study, we aim to minimize user operation and device maintenance.

Protecting user security and privacy has been a topic of active research in smart homes. In [46], an architecture for a healthcare system in a smart home has been introduced. Sokullu et al. [47] present an IoT-based smart home system for elderly people and people with partial memory loss. In [48], a health-smart home system that uses a renewable source of energy for monitoring was proposed. Talal et al. [49] present an established IoT-based smart home security solution for real-time health monitoring systems. These studies have contributed significantly to protecting the security and privacy of users at home, especially for the elderly.

## 3. Methodology

### 3.1. Preliminary Study

**Figure 1:**
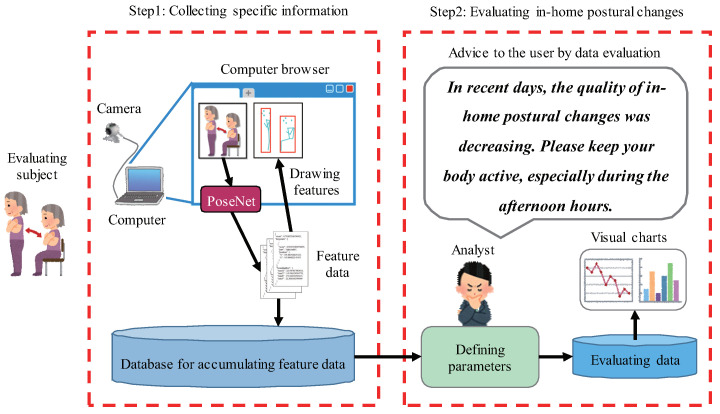
The overall architecture in the preliminary study.

**Figure 2:**
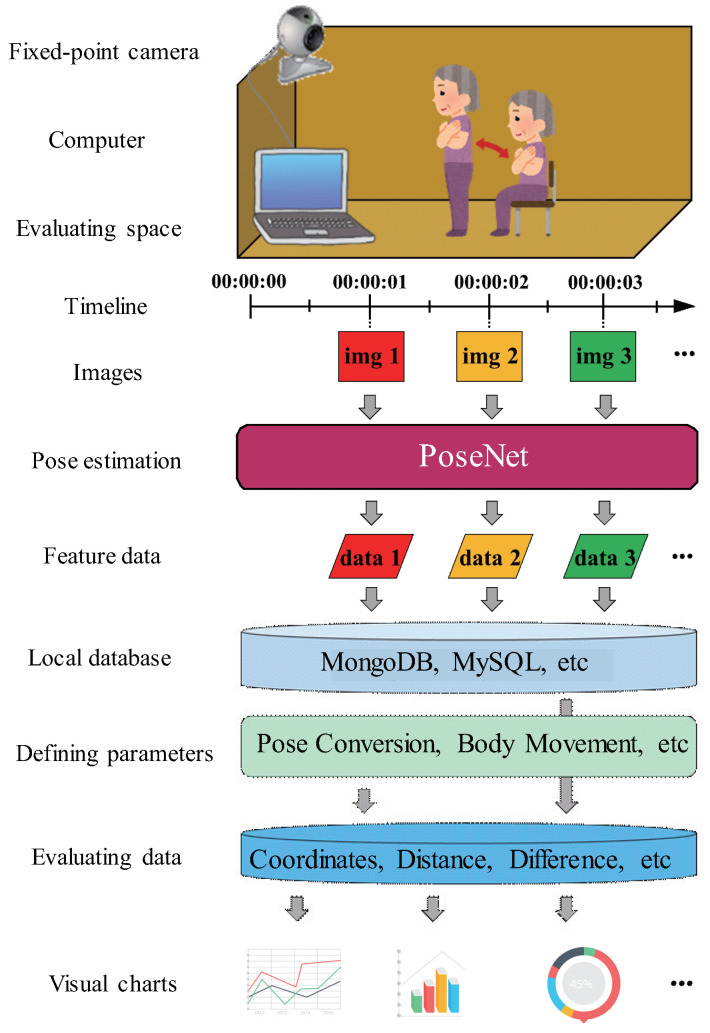
Flowchart of the proposed method in the preliminary study.

Extracting and evaluating feature data is a basic and indispensable part of realizing continuous home care monitoring. The key step of evaluating feature data is to define the evaluation parameters and scales, which makes feature data more meaningful for individuals. This section presents a preliminary implementation of nonintrusive home care monitoring by characterizing the quality of in-home postural changes with a computer browser. Figure 1 shows the overall architecture in this preliminary study. Unlike conventional approaches, we use a standalone model of pose estimation to extract feature data of the person requiring care in a local process. For this, we present a creative evaluation method that includes a current pose map drawn in the browser, to generate the related charts by manually calculating the feature data. The flowchart of the proposed method is shown in Figure 2. The procedure comprises the following two major steps:

**Step 1: Collecting feature data using the computer browser**

**Figure 3:**
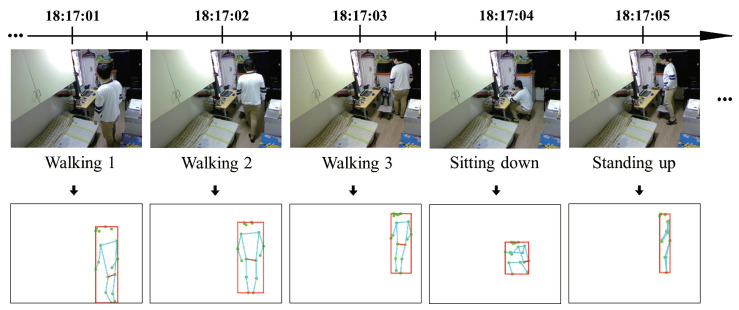
Example of the pose maps representing postural changes in the timeline.

**Step 1-1: Receiving images by time series**
  We first install a device that can capture images (e.g., USB camera) in a room where the person requiring care is located. Then, we connect the device to a laptop or desktop computer. Next, we use the computer to create HTML and JavaScript files as in [50,51] for embedding live video to the computer browser (we recommend using Google Chrome).
**Step 1-2: Extracting feature data with a PoseNet model**
  We first extend the functions of Step 1-1 to receive every image of the live video for definite time intervals (e.g., one second), and draw it on a canvas with a fixed resolution (refer to [52]). Then, we extract the feature data of each canvas using a PoseNet model that can be called with the browser [22,53]. In recent years, other pose estimate models such as the ones described in [54,55,56] have demonstrated potential for use in smart homes. To improve the process of continuously extracting feature data, we introduce a method to implement an offline PoseNet model. For this, we obtain the calling link of an online PoseNet model and download the related model files, including two algorithms with “MobileNet” and “ResNet50” [22], by inspecting the network activity of the browser (refer to [57]) when running a process of extracting the feature data. Finally, to convert the paths of the model files to a local link that can be used instead of the original calling online link, we create a local web server using the live-server of Node.js [58]. In this manner, we realize a local process of extracting the feature data of the person requiring care.
**Step 1-3: Accumulating feature data in a local database**
  We first create a local database (refer to [59,60]) that can accumulate feature data, such as MongoDB [61] and MySQL [62]. Then, we post each extracted feature data as a time series to the local database using an Ajax technique [63] in the related Javascript file. Moreover, we use the Javascript code to draw each extracted feature data in the canvas (refer to [22]) that can represent and automatically update each pose map, shown in Figure 3.

**Step 2: Evaluating the quality of in-home postural changes**

**Figure 4:**
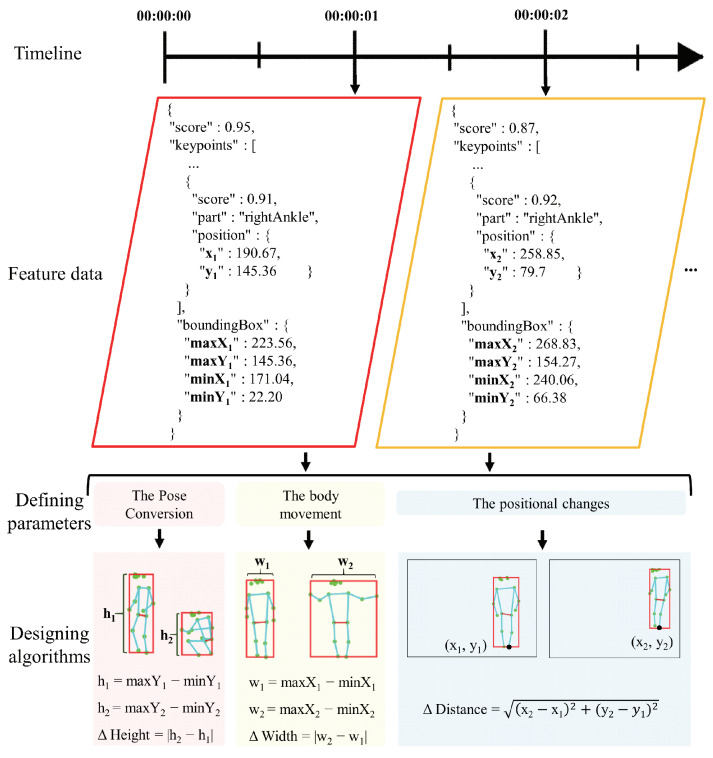
Example of feature data, defining parameters and designing algorithms within the timeline.

**Step 2-1: Defining parameters to be evaluated**
  We define *m* parameters as $I=\{i_{1},i_{2},...,i_{m}\}$, to evaluate specific cases for users. For example, to characterize the quality of in-home postural changes for a person requiring care, it is possible to firstly define the changes in the current pose, including the conversion among standing up, sitting down, and walking at home, the parameters to be evaluated.
**Step 2-2: Designing algorithms to obtain the evaluated data**
  We first set a time period to accumulate the feature data. Then, for each parameter $i_{j}$ ($i_{j}\in I$, 1 ≤ *j* ≤ *m*) to be evaluated, we manually analyze and extract the required data from the local database of Step 1-3. Then, we calculate data with tools such as Microsoft Excel. We aim to characterize the quality of in-home postural changes by providing the evaluating data. These are usually included in fine-grained variables of the body, such as the pose conversion, body movement, and positional changes. We consider that these parameters are linked with the changes in the shape and the positional changes of the pose bounding box. Our key idea is to use the feature values of the pose bounding box to calculate changes in the width, height, and distance (e.i. ΔHeight, ΔWidth, and ΔDistance in Figure 4) for different poses.
**Step 2-3: Evaluating data by the visual charts**
  We combine the data calculated in Step 2-2 with the timeline, and generate the related charts to analyze each evaluation parameter $i_{j}$ within the period. Specifically, for each parameter $i_{j}$ ($i_{j}\in I$, 1 ≤j≤m) to be evaluated, we regard the average value of the changes per minute as the evaluating data on the time series. Then, we generate charts with tools such as Microsoft Excel. In this manner, we can offer timely advice to the user by evaluating the defined parameters within the period.

### 3.2. Proposed Method

**Figure 5:**
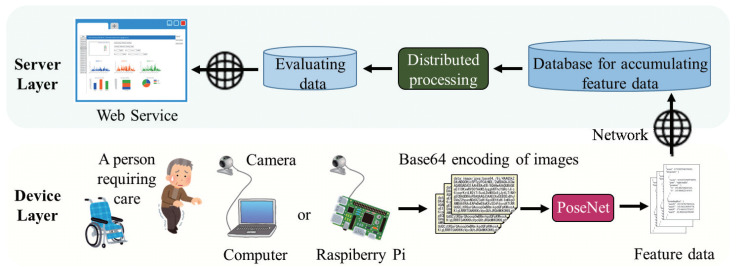
Flowchart of the proposed method.

**Figure 6:**
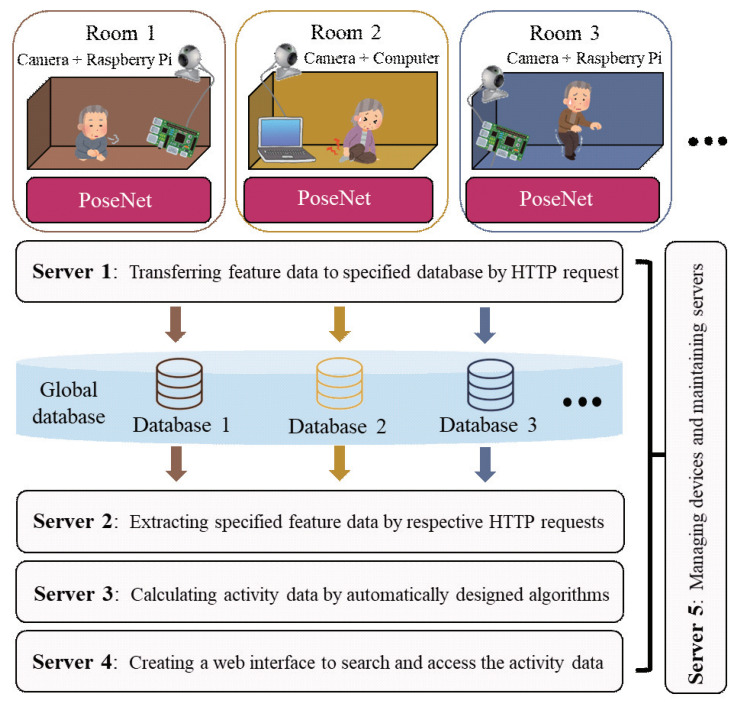
The architecture of distributed processing with multiple servers in the proposed method.

The preliminary study presents a local process of continuously extracting feature data. However, the process requires large amounts of the cache memory when a continuous process with the computer browser. Moreover, the extracted feature data cannot be flexibly customized to automatically generate the required data to be evaluated. The flowchart of the proposed method is shown in Figure 5. In this section, we introduce a fine-grained home care monitoring system for different households. We extend the extraction of feature data from the computer browser to Raspberry Pi, and use multiple servers to realize the distributed processing for deploying home monitoring as a web service. Figure 6 shows the architecture of distributed processing with multiple servers in the proposed method. We optimize the process based on the preliminary study, mainly by adding the following three major steps:   

**Step 3: Collecting feature data using a Raspberry Pi**

**Step 3-1: Running a loop for receiving image Base64 encoding**
  We first connect a camera device to a Raspberry Pi [27], preferably above version two, to ensure optimum performance in this step. Then, we create a separate javascript (i.e., Node.js [64]) file. To run a loop for receiving image Base64 encoding once every second, we use the package “OpenCV” (refer to [65]). A new process can be run at each time step key to maintaining the loop function continuously.
**Step 3-2: Drawing the image Base64 encoding on a canvas**
  We first extend the functions of Step 3-1 to receive every image Base64 encoding once every time interval (e.g., one second), and draw it on a canvas (the package refer to [66]). Please note that we maintain the same resolution for the image Base64 encoding and the created canvas.
**Step 3-3: Extracting feature data and disposing memory**
  We first extract feature data from each canvas using the packages in “@tensorflow”, including “@tensorflow/tfjs-node” [67] and “@tensorflow-models/posenet” [22]. Listing 1 shows an example of extracting feature data and disposing memory with Node.js. To ensure accuracy of the PoseNet model, the related parameters and algorithms (e.g., “architecture”, “outputStride”, and “multiplier”) can be flexibly chosen and as per individual requirements (refer to [53]). Furthermore, we can also set a threshold value to exclude a part of data that overall low accuracy.

Listing 1 **Example of extracting feature data and disposing memory with Node.js.**

const tf = require("/node_modules/@tensorflow/tfjs-node");

const posenet = require("/node_modules/@tensorflow-models/posenet");

const net = await posenet.load();

async function heavyTask(net) {

  tf.setBackend(’tensorflow’);

  tf.engine().startScope();

  var tensor = tf.browser.fromPixels(canvas);

  var pose = await net.estimateSinglePose(tensor);

  console.log(pose);

  tensor.dispose();

  tf.engine().endScope();

};

await heavyTask(net);

**Step 4: Generating the required data for evaluation**

**Step 4-1: Transferring feature data to specified database by HTTP request**
  We apply the database client package into a standalone server, such as in [68,69,70], to post feature data at each time interval. Specifically, we first set the host IP address of the database to the specific URL. Then, we use a package called “express” to build a REST API, which is linked with the database (refer to [71,72]).
**Step 4-2: Extracting specified feature data by respective HTTP requests**
  We apply the same database client package as in Step 4-1 to a different server to obtain feature data at any time. We employ this step due to the post data caused by devices, and to obtain data from web service calling (refer to Step 5).
**Step 4-3: Calculating activity data by automatically designed algorithms**
  We apply the feature data from Step 4-2 to the designed algorithms (refer to Figure 4). Specifically, each algorithm is developed as a function into the related files to calculate the activity data automatically.

**Step 5: Deploying home care monitoring as a web service**

**Step 5-1: Creating a web interface to search and access the activity data**
  We organize the responses of the distributing process to create the web user interface (UI) with the corresponding embedded JavaScript (EJS) templates [73], to implement functions that include searching data from specific time points to generate pose maps and visual charts (refer to [74,75]). Moreover, the total time hours of postural changes for each day can be calculated based on the amount of posting data.
**Step 5-2: Managing devices and maintaining servers**
  To avoid ambiguities in interpreting data and ensure continued data access for all users, we first monitor and manage the continuity of the posting data by each device. Then, we check if the device is online, by comparing the time of the latest data with the current time. To avoid errors in the program, we incorporate exception handling in each process. Then, we bind a URL to each server separately. In this manner, we set a server dedicated to monitoring the status, and use the package “url-exist” [76], to monitor the validity of the URLs of each server in real time.

### 3.3. Discussion

Using PoseNet model for recognizing the specific poses have been presented in [77,78,79]. However, in pose estimation for home care monitoring, there are cases where multiple people may be present. Data may be misinterpreted by the simultaneous appearance of other persons in the fixed-point home space. To address this issue, we introduce two modules for users to choose from: (1) The misinterpreted data can be used as the characteristic data of the variation of daily home care in single-person pose estimation. By visualizing the time variance of home care, the details of daily life and living habit can be objectively evaluated. This can serve as reference data to check regularly in long-term home care. (2) The misinterpreted data can be excluded limiting the number of outputted datasets in multi-person pose estimation. In this case, the system will only record the status of in-home postural changes when family caregivers are not attending to the person requiring care.

From the pose maps shown in Figure 3 and Figure 4, we extract and draw all feature values outputted by the PoseNet model. The primary between this study and existing methods is that we regard the feature values of the pose bounding box instead of the 17 body keypoints as the evaluation data. Gavrilyuk et al. [80] use the feature values of the pose bounding box to crop images of group activity to single-person images, to compare pose differences. This can be used to improve the results of our study. In [81], an insight has been provided into how the body motion and action features from views of two cameras are correlated. A similar study [82] described the 3D pose map. We will refer to their method to extend this study using multiple cameras. In [83], pose estimation is classified using the feature values of the pose bounding box. We consider that it is inapplicable to home care monitoring systems that require time to build models with respect to individual and environmental differences.

## 4. System Evaluation

### 4.1. Accuracy of Evaluating Data

To test the correct rate of in-home postural changes that are required to evaluate the accuracy of the bounding box outputted by the PoseNet model in the proposed system. For this, we have downloaded the Leeds Sports Pose (LSP) dataset, which contains 2000 single-person images and a mat file describing the coordinates of the correct body keypoints position for each image [84]. The downloaded images were all scaled versions. According to [85], the most prominent person is roughly 150 pixels in length.

Our purpose is to evaluate the outputted results of two architectures (i.e., MobileNet, ResNet50) in the PoseNet model. For this, we developed a program that uses different architecture to output the result of every image once a second in the proposed system. At the same time, we have obtained the maximum and minimum values of X and Y for each correct coordinate in the LSP images. Next, we have applied the maximum and minimum values of X and Y for each image of MobileNet, ResNet50, and LSP into the designed algorithm (refer to Step 2-2 in Section 3 and Figure 4).

Table 2 shows the percentage of total images in every difference range by putting the results of PoseNet two architectures and LSP into the designed algorithms to compare respectively. The difference value here is actually the difference in the outputted pixel coordinates. From Table 2, we found that when the difference value is within 10, the correct rate of ResNet50 was stable at about 70%, which is greater than that of MobileNet. Moreover, the highest and lowest correct rates are the changes in width and distance respectively. The magnitude opposite to the above description appears if the difference value is between 10 and 20. The correct rate of MobileNet was about 12% and the ResNet was about 7% if the difference value is greater than 30.

### 4.2. Usage Status of System Memory and CPU

**Figure 7:**
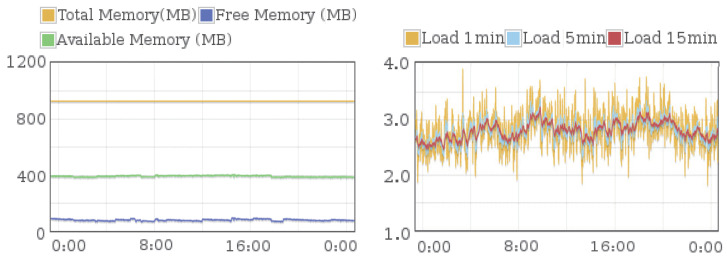
Usage status of system memory and CPU that collecting feature data using a Raspberry Pi.

To monitor the process of collecting feature data in 24 hours, we use the computer (CPU: AMD Ryzen 5 3500U, RAM: 8G) browser that was Google Chrome (version 84.04147.125), and the Raspberry Pi used that was (3 model B, GNU/Linux 8.0 (Jessie)). The status of system memory and CPU within 24 h were monitored. From the results of Google Chrome, we observed that the usage of system memory and CPU respectively was 50% and 10%. The results that collecting feature data using a Raspberry Pi is shown in Figure 7. From this, it can be seen that the usage of system memory and CPU respectively was around 30% and 70%. From the above results, we found that the memory consumption of collecting data with Raspberry Pi was lower than that of computers. However, its CPU occupancy rate was higher than that of computers. In this test with 24 h, the system memory and CPU both showed the stability of the variation range.

## 5. Actual Experiment

### 5.1. Experimental Setup

**Figure 8:**
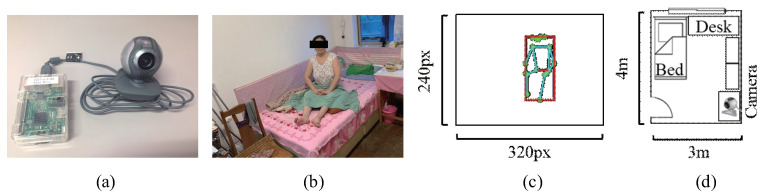
Experimental Setup: (**a**) A Raspberry Pi and a USB camera. (**b**) The experimental room and the elderly requiring care. (**c**) An example of the pose map with the set resolution. (**d**) The environmental layout of target space.

Figure 8 shows images of the USB camera, Raspberry Pi, experimental room, the elderly requiring care, an example of the pose map with the set resolution, and the environmental layout of the experimental single-room. Environmental and application settings are shown in Table 3. Please note that when the feature data cannot be extracted due to conditions such as the lights being turned off or the absence of the person in the room, we ensure that an empty dataset is not automatically uploaded to the servers. Every visual chart shows the average value for every minute using the method of the simple moving average (SMA) [86].

### 5.2. Results

**Figure 9:**
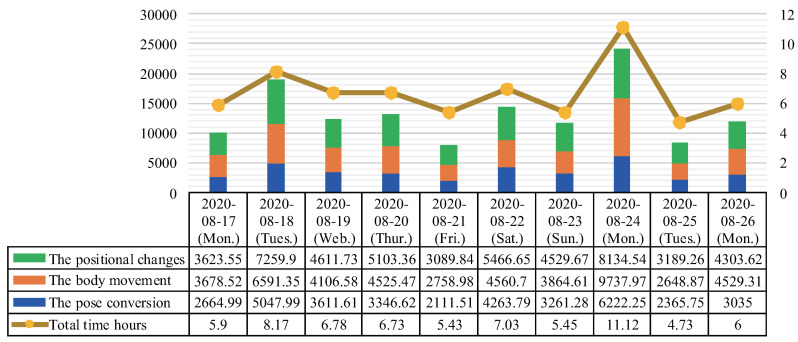
Cumulative results of all parameters and total time hours on each day.

Figure 9 shows the cumulative results of all parameters and total time hours on each day via a combination chart. The horizontal axis of this chart represents experimental dates, the vertical axis on the left represents the coordinate variables, and the vertical axis on the right represents the total time hours of postural changes per day. It can be seen that due to the physical discomforts of the person requiring care, the pose conversion is always smaller than the body movement and positional changes. Then, we found that the value of the body movement on 24 August exceeded the positional changes, indicating that more activities than usual on that day. Since the total results on 24 August was higher than usual, the total results on 25 August dropped significantly. In addition, we can see that the highest number of in-home postural changes were performed on 18 August and 24 August. We found the following three trend changes. (1) The results of postural changes increased from 17 to 18 August. (2) During the period from 19 to 21 August, the results of postural changes tend to decrease. (3) The results of postural changes during the period from 22 to 26 August showed an unstable trend. From these trends in the changes, family caregivers could adjust the frequency of home care to maintain the health of the subject in a timely manner. Furthermore, if changes in the pose conversion and the positional changes tend to be greater than the body movement, it can be seen that the subject recovers effectively. From the results, we found that 24 and 25 August are the days with the most and least total time hours, respectively. By taking the average of the total time hours of the remaining days, we advise that the subject should limit the time of in-home postural changes to about six and a half hours every day. Moreover, to prevent the subject from getting tired, we estimate, based on the waking time, lunch break time, and sleep time, that it is best not to spend more than three hours for physical rehabilitation training every day.

**Figure 10:**
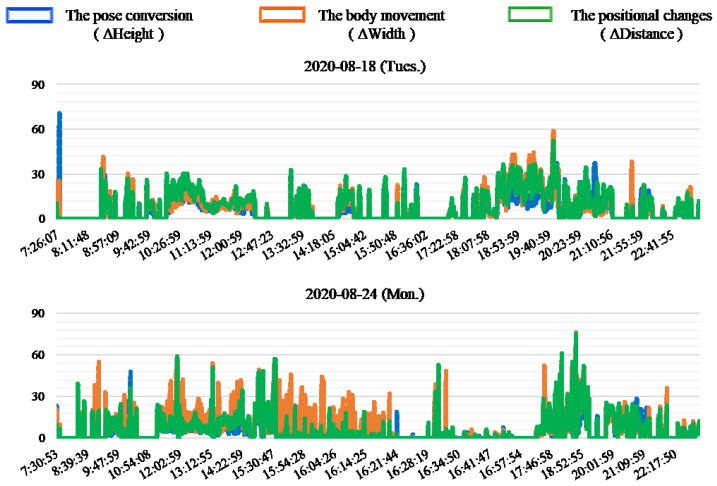
Example of the results of the typical two days in this experiment.

**Figure 11:**
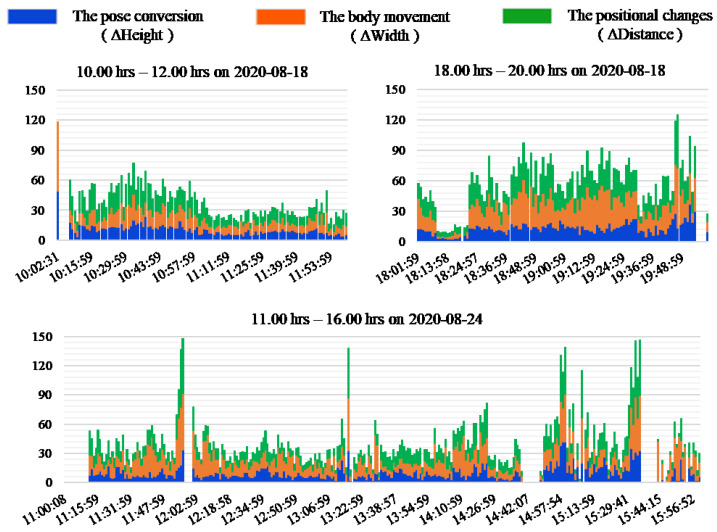
Example of the results with continuous postural changes in this experiment.

Figure 10 shows an example of the results of the typical two days in this experiment. Figure 11 shows an example of the results with continuous postural changes in this experiment. The horizontal axis of each chart represents the timeline, and the vertical axis represents the average value of coordinate variables per minute. When the change value is zero on the same period, it can be seen that the pose conversion and the body movements are the same as that during the positional changes. This shows that any postural changes with respect to these three aspects. The results also show that there is a continuity in the changes in certain specific periods. In Figure 11, from 10:00 to 12:00 and 18:00 to 20:00 h on 18 August, and from 11:00 to 16:00 h on 24 August. In these cases, we consider that feature data of multiple people are present, which include family members and doctors. Furthermore, in most of the results, it can be seen that changes in pose conversion are smaller than that in the body movement, but changes in the body movement are greater than that in the positional changes. This can be attributed to the discomfort in the legs and feet of the subject, to show more activities of the hands. Furthermore, we found that the subject took a lunch break around 16:00 h on most days, including from August 17 to 22 and 25 August, although the duration of these breaks were different. Moreover, from the analysis of the waking time, it can be seen that the subject woke up 08:00 h every day, except on 22 and 23 August. In addition, from the sleep time of the results, it also can be seen that the sleep time on 22 and 23 August is around 01:00 h.

### 5.3. Discussion

Figure 9 reflects the relationship between the times and number of postural changes to a certain extent. It can be further seen that the number of postural changes is the smallest on 21 August, and the largest on 18 August and 24 August if the number of postural changes is divided by the activity times. Figure 10 and Figure 11 show that the postural changes of each parameter in different periods. In particular, we can see that the parameters at different time points with the highest number of postural changes in Figure 11. The most important data is not only the number of postural changes but also the continuous postural changes. For example, the data from 11:00 to 16:00 h on 24 August showed that most of the body movement was greater than the positional changes, and the consistency of postural changes have shown. In this case, we believe that home care is in progress. In other words, the subject data contains caregiver data. In this manner, we can deduce that in days other than 18 August and 24 August, the general care times and frequency are not much. Therefore, we recommend that the subject take regular home care every day to promote physical recovery. Correlating Figure 10 and Figure 11 with Figure 9, we found that the presence of caregivers increases the number of in-home postural changes of the subject. The elderly may otherwise be unwilling to perform postural changes on their own. Moreover, continuous home care for prolonged periods causes physical and mental exhaustion. To address this, we will analyze changes in various mental and physical functions with in-home postural changes in future work.

## 6. Conclusions

In this paper, a technique that integrates inexpensive camera devices and bone-based human sensing to implement a nonintrusive fine-grained home care monitoring is described. The effectiveness of the proposed model can be established from the comparison of the experimental evaluation, the daily habits of the elderly requiring care, irregular home care timings, and the changes in a series of postural changes caused by external factors.

Home care monitoring is of great significance for grasping the postural changes of people requiring care at home. However, the acquisition and differentiation of feature data for multiple people are difficult issues to be addressed fundamentally. A drawback of the proposed method lies in the lack of detailed descriptions of the home care process of multiple people. Regarding the PoseNet model with a function of multiple-person pose estimation, the most difficult task is to distinguish the properties of feature data. In this manner, recording the feature data of a single-person when family caregivers are beside the subject may be considered imprecise. In the experiment of this paper, the accounts for the data misinterpretation caused by the presence of experiment with multiple people when the single-person pose estimation is used, and precisely reflects the duration of the home care. We consider that the health status of family caregivers also changes the effect of home care, indirectly influencing the health status of the person requiring care at home. Therefore, it is also necessary to grasp the health status of family caregivers in future work. In addition to using existing data, we plan to consider the suggestions of hospitals and facilities to establish smart home care with the remote professional guidance in the future.

References yes

## Figures and Tables

**Table 1:** A comparison of related work for home monitoring.

Research Authors (Year)	Monitoring Target	Materials and Methods	Intrusiveness	Time Intervals of Data Acquisition
Nilpanapan et al. [28] (2016)	The gait behaviors	Social data shoes that include five force sensitive resistors (FSRs)	Exist	One second
Hossain et al. [29] (2016)	The patient status	Video cameras and microphones, multimodal inputs, a dedicated cloud	Exist	None
Guan et al. [30] (2017)	The heart rate	Wearable smart clothing, home gateway, health care server	Exist	Six seconds
Chiridza et al. [31] (2019)	The risk and safety of the elderly living independently	A Raspberry Pi, a Microsoft Kinect sensor and an Aeotec 4-in-1 Multisensor	None	One hour
Our research in this paper (2020)	The postural changes	Camera Devices, bone-based human sensing technologies, web servers, a Raspberry Pi	None	One second

**Table 2:** The percentage of total images in every difference range by putting the results of PoseNet two architectures and LSP into the designed algorithms to compare respectively.

Difference Value (K)	PoseNet (MobileNet)–LSP Results			PoseNet (ResNet50)–LSP Results		
	ΔHeight	ΔWidth	ΔDistance	ΔHeight	ΔWidth	ΔDistance
K ≤ 10	62.55%	56.45%	65.80%	71.50%	69.40%	77.60%
10 < K ≤ 30	28.00%	31.95%	25.60%	21.80%	24.20%	17.40%
K > 30	9.45%	11.60%	8.60%	6.70%	6.40%	5.00%

**Table 3:** Environment and application settings.

Target space	Single-room (4 m × 3 m)
Experimental period	10 days (17 to 26 August, 2020)
Evaluated subject	An aged woman (recovering from a broken leg)
Shooting device	USB camera (Logitech OEM B500)
Shooting position	In a corner of the room (Figure 8d)
Shooting interval	1 s
Image resolution	320 × 240
Application device	Raspberry Pi 3 Model B
Pose estimation model	PoseNet model
Pose estimation type	Single-person pose estimation
Model architecture	ResNet50
Pose estimation threshold	0.5
Number of defined parameters	3 (refer to Figure 4)
